# DHOEM: a statistical simulation software for simulating new markers in real SNP marker data

**DOI:** 10.1186/s12859-015-0830-7

**Published:** 2015-12-03

**Authors:** Laval Jacquin, Tuong-Vi Cao, Cécile Grenier, Nourollah Ahmadi

**Affiliations:** CIRAD, UMR AGAP, Centre de Coopération Internationale en Recherche Agronomique pour le Développement, Avenue Agropolis, Montpellier Cedex 5, 34398 France

**Keywords:** Data simulation, Data structure, Likelihood, Non-parametric, LD, Haplotypes, SNP, Genomic relationship matrix, Minor allele frequency

## Abstract

**Background:**

Numerous simulation tools based on specific assumptions have been proposed to simulate populations. Here we present a simulation tool named DHOEM (densification of haplotypes by loess regression and maximum likelihood) which is free from population assumptions and simulates new markers in real SNP marker data. The main objective of DHOEM is to generate a new population, which incorporates real and simulated SNP by statistical learning from an initial population, which match the realized features of the latter.

**Results:**

To demonstrate DHOEM’s abilities, we used a sample of 704 haplotypes for 12 chromosomes with 8336 SNP from a synthetic population, used for breeding upland rice in Latin America. The distributions of allele frequencies, pairwise SNP LD coefficients and data structures, before and after marker densification of the associated marker data set, were shown to be in relatively good agreement at moderate degrees of marker densification. DHOEM is a user-friendly tool that allows the user to specify the level of marker density desired, with a user defined minor allele frequency (MAF) limit, which is produced in a reasonable computation time.

**Conclusions:**

DHOEM is a user-friendly and useful tool for simulation and methodological studies in quantitative genetics and breeding.

## Background

Simulation studies have become a popular cost effective approach to assess both new methods for statistical analysis [1] and the power of experimental designs [2]. For example, simulating populations with a large number of SNP markers can be a useful way to evaluate new statistical methods for genome wide association studies (GWAS) or genomic selection (GS). The many existing softwares for genetic data simulation can be classified under three main approaches [3]: coalescent [4–6], forward-time [7–9] and re-sampling [10–12]. However, some of these simulation approaches are often based on specific population assumptions (effective population size, mutation rate, bottlenecks, etc..) which can lead to substantial deviations from the realized features, i.e. the linkage disequilibria (LD) and allele frequencies, of a target population. For example, simulating populations forwards as suggested in [13], or backwards in time as suggested in [14], does not take into account available observed genetic data and can struggle to match real LD patterns [15], especially in populations whose evolutionary history cannot be ascertained.

As pointed out in [16], there are so many different forms of genomic variability and population histories that it is impossible to propose a single correct model for simulating data. As described in [16], one reasonable way to overcome these limitations consists in matching the realized features of simulated data with those of observed data. Hence some simulation tools have been based on re-sampling, of observed data from reference panels, to overcome the limitations of forward and backward approaches [15]. Nevertheless the number of SNP markers simulated with a re-sampling approach is usually limited to that of a reference panel [3], in contrast to forward or backward approaches where theoretically no such limitation exists. What is more, reference panels are not available for all species, and the individuals included in these panels have to be representative of the population being studied, otherwise the realized features of the simulated population may deviate substantially from those of the target population under study.

Here we present a new simulation software named DHOEM (which stands for densification of haplotypes by loess regression and maximum likelihood) that does not belong to the three aforementioned approaches. DHOEM is a statistical procedure that simulates new markers, according to statistical modeling of local data characteristics, in real SNP marker data sets. The main objective of DHOEM is to increase the marker density in a marker data set for simulation studies. For each chromosome in a marker data set, the statistical procedure defined in DHOEM models the probability distribution generating the allele frequencies and the relation between LD and physical distance between consecutive markers.

To some extent, DHOEM resembles imputation methods used to increase marker density in an existing marker data set based on a reference panel. However, DHOEM does not require a reference panel since it simulates new markers only according to a statistical procedure. In addition, unlike DHOEM, imputation methods are not intrinsically simulation softwares. Yet, they can be another strategy for increasing marker density for simulation studies, although the concordance of the imputed markers, with respect to allele frequencies and LD in the marker data set, will depend on the available reference panel [17]. In this paper we used a synthetic population for breeding upland rice in Latin America, to demonstrate DHOEM’s abilities and to briefly compare them with those of BEAGLE 4.0 [18] (http://faculty.washington.edu/browning/beagle/beagle.html) for simulation purposes.

## Implementation

In this section we describe the implementation of the statistical procedure and the modeling and optimization routines defined in DHOEM. The software is written in R and runs on Windows operating systems (OS), although with a few modifications, it can be extended to Linux-like OS.

### Statistical procedure defined in DHOEM

Suppose we have *N* haplotypes, defined for a set of *L* distinct chromosomes, such that each of them is composed of *P*_*j*_ SNP markers with *j*∈{1,..,*L*}. Further assume that the physical distances between markers are in Kilobases (Kb). Let $Z^{(i)}_{\textit {jk}} \in \lbrace 0,1 \rbrace $ be the random variable associated to the realized allele $z^{(i)}_{\textit {jk}}$ at marker *M*_*jk*_ (*k*∈{1,..,*P*_*j*_}) for any haplotype *i* (*i*∈{1,..,*N*}). Let *X*_*jk*_ denote the random variable associated to the realized allele frequency *x*_*jk*_ at any marker *M*_*jk*_. The statistical procedure for simulating new markers on chromosome *j* is defined by two steps:

#### Step 1 (learn the processes generating the data):

- 1.1 The observed allele frequencies at markers are modeled by a beta distribution: *X*_*jk*_∼*B* *e* *t* *a*(*α*,*β*), where the estimated values $\hat {\alpha }$ and $\hat {\beta }$ for the shape parameters are obtained by minimizing the associated negative log-likelihood objective function using a *descent direction* algorithm.
- 1.2 The absolute correlation *ρ*(*Z*_*jk*_,*Z*_*jk*+1_) (i.e. LD) between any two consecutive markers *M*_*jk*_ and *M*_*jk*+1_, for all haplotypes, is modeled as a loess regression function *f*(.)_*λ*_ of the physical distance $d_{M_{\textit {jk}},M_{jk+1}}$ between the markers.

#### Step 2 (simulate from the learned processes):

- 2.1 The realized allele frequency *x*_*j*∗_ for a marker *M*_*j*∗_ simulated between *M*_*jk*_ and *M*_*jk*+1_ is sampled from $Beta(\hat {\alpha },\hat {\beta })$.
- 2.2 The physical distance $d_{M_{\textit {jk}},M_{j*}}$ of *M*_*j*∗_ from *M*_*jk*_ is sampled from a continuous uniform distribution $\mathcal {U}$ on $]0, d_{M_{\textit {jk}},M_{jk+1}}[$. The required correlation between *M*_*j*∗_ and *M*_*jk*_ is then predicted by $\hat {\rho }_{jk*}=\hat {f}\left (d_{M_{\textit {jk}},M_{j*}}\right)_{\lambda }$.
- 2.3 A temporary vector $V_{j*}=\left [z_{j*}^{(1)},..,z_{j*}^{(i)},..,z_{j*}^{(N)}\right ]$ of realized alleles at *M*_*j*∗_ is generated by sampling from a Bernoulli distribution $\mathcal {B}$ with parameter $\phantom {\dot {i}\!}x_{j^{\ast }}$ (i.e. $Z_{j\ast }^{(i)} \sim \mathcal {B}(x_{j\ast })$). The vector *V*_*j*∗_ is then transformed into a vector $\tilde {V}_{j*}$ such that $\rho \left (Z_{\textit {jk}},\tilde {Z}_{j*}\right)=\hat {\rho }_{jk*}$, under the constraint $\tilde {x}_{j*}=x_{j*}$, by solving the following equation for the expectation of the product $Z_{\textit {jk}}\tilde {Z}_{j*}$:
  $${} {\fontsize{9.2pt}{9.6pt}\selectfont{\begin{aligned} \mathbb{E}\left[ Z_{jk}\tilde{Z}_{j*} \right]&=\sqrt{Var\left(Z_{jk}\right)}\sqrt{Var\left(\tilde{Z}_{j*}\right)} \times \rho\left(Z_{jk},\tilde{Z}_{j*}\right) \\ &\quad+ \mathbb{E}\left[Z_{jk}\right]\mathbb{E}\left[\tilde{Z}_{j*}\right] \\ &= \sqrt{x_{jk}\left(1-x_{jk}\right)}\sqrt{x_{j*} \left(1-x_{j*}\right)} \times \hat{\rho}_{jk*} + x_{jk}x_{j*} \end{aligned}}} $$
  Subsequently, $N.\mathbb {E}\big [ Z_{\textit {jk}}\tilde {Z}_{j*} \big ]$ gives the required number of co-occurrences of allele 1 at *M*_*jk*_ and *M*_*j*∗_, under the constraint $\tilde {x}_{j*}=x_{j*}$, such that $\rho (Z_{\textit {jk}},\tilde {Z}_{j*})=\hat {\rho }_{jk*}$. The vector $\tilde {V}_{j*}$ is finally returned as the vector of realized alleles at *M*_*j*∗_.

### Modeling and optimization routines defined in DHOEM

Modeling the observed allele frequencies on each chromosome by a beta distribution is a natural choice for populations verifying panmixia, and/or under selection, since the distribution shape (concave, convex, etc.) can change depending on the values of $\hat {\alpha }$ and $\hat {\beta }$. The *descent direction* used to minimize the associated negative log-likelihood objective function of the beta distribution, at each iteration, is given by the Broyden-Fletcher-Goldfarb-Shanno (BFGS) update multiplied by the negative gradient of the objective function [19].

It should be recalled that a *descent direction* for a multivariate differentiable function, evaluated at some point in space, is a direction vector that minimizes the *directional derivative* of the function at that point, i.e. this vector gives a direction along which to move such that the objective function can decrease. Since the *directional derivative* corresponds to the inner product between the gradient of the objective function and a direction vector, the negative gradient is often used to define a *descent direction* as it minimizes this inner product.

The BFGS update at each iteration is a positive definite approximation of the Hessian matrix of the objective function, based on accumulated information from the gradients and inputs in previous iterations, which enables a very high convergence speed of the descent algorithm [19].

Modeling LD as a non-parametric loess regression function of the physical distance, on each chromosome, is based on the fact that there is an unclear relationship between LD and physical distance that can vary with chromosomal location [20]. Indeed, one can often observe a high variability of LD locally, as a function of the physical distance between pairwise biallelic markers, which can be accounted for by loess regression. The smoothing parameter *λ* for the triweight kernel function used in loess is evaluated using *K*-fold cross-validation with *K*=3 to limit computation time. The loess.wrapper(.) and optim(.) functions, from the bisoreg [21] and stats packages, are used to respectively implement the loess regression and a limited memory and bound constraint version of the BFGS algorithm [22].

## Data sets, imputation and simulation

In this section we describe the marker data set used to compare DHOEM with BEAGLE 4.0, and respectively the imputation and simulation done with the two approaches.

### Data sets

The marker data set used was composed of 704 haplotypes with 8,336 SNP for 12 chromosomes. It came from a synthetic population used for breeding upland rice in Latin America [23]. The data set had no monomorphic markers and 7,879 SNP had a minor allele frequency (MAF) ≥ 1 %. A reference panel for this population, composed of 334 haplotypes with 16,444 markers (6,717 SNP + 9,727 DArT) as described in [24], was used for imputation of the marker data set with BEAGLE 4.0. All 16,444 markers in the reference panel had a MAF ≥ 1 %. The reference panel and the marker data set had 4,015 SNP markers in common. Two individuals in the reference panel, out of a total of 167, shared recent common ancestry with the 352 individuals associated with the marker data set [23, 24].

### Imputation with BEAGLE 4.0

BEAGLE 4.0 was used to increase marker density in the marker data set up to 16444. The parameter values used for imputation with BEAGLE 4.0 were *i* *m* *p* *u* *t* *e*−*i* *t* *s*=10, *w* *i* *n* *d* *o* *w*=300 and *o* *v* *e* *r* *l* *a* *p*=150. The parameter *i* *m* *p* *u* *t* *e*−*i* *t* *s* controls genotype imputation accuracy and was set to 10 for highest imputation accuracy, according to BEAGLE 4.0 documentation; http://faculty.washington.edu/browning/beagle/beagle.29Sep14.pdf. The parameters *window* and *overlap* respectively control the amount of memory used in the analysis and specify the number of markers of overlap between sliding windows. As suggested by the authors, the value of the *window* parameter was chosen to be at least twice as large as the *overlap* parameter. Following the recommendations in the BEAGLE 4.0 documentation, the *overlap* parameter was set to 150, according to the marker densities of the data sets.

### Simulation with DHOEM

The following single line command was used to call DHOEM for the densification of the marker data set to at least 16,444 SNP with MAF ≥ 1 %. The marker data set is provided with the simulation software and is composed of the three.txt input files in the command.



The parameter User_Name in the command is the current user name in any Windows environment. The second parameter corresponds to the average length (in Kb) of the regions around centromeres with low SNP coverage. The three last parameters control the MAF limit, the number of SNP added to (or removed from) the marker data set, at each run of the program, and the desired minimum (or maximum) marker density after densification (or loosening) of the marker data set. The MAF limit parameter is not involved in the statistical procedure defined in DHOEM. This parameter only assures that the MAF of the output markers are greater or equal to the defined limit. The computation time for this command is 3 to 4 minutes on a personnal computer with 4 cores (16 GB RAM). Note that the combination of explicit parameter names in this single line command constitutes a user-friendly framework.

## Results and discussion

In this section, we describe the data generated by BEAGLE 4.0 and DHOEM and discuss the relevance of these data for simulation studies. The limits and advantages of DHOEM are also discussed.

### Description and relevance of the data generated by BEAGLE 4.0 and DHOEM

The imputed marker data set obtained with BEAGLE 4.0 had 16,444 markers. The reference panel and the marker data set had only 4,015 SNP markers in common. Hence only 4,015 SNP markers from the data set were found in the imputed data set since BEAGLE 4.0 always excludes variants that are absent from the reference panel (see BEAGLE 4.0 documentation). Only 9,154 out of the 16,444 markers from the imputed marker data set had a MAF ≥ 1 %, and only 10,105 out of the 16,444 had a MAF ≥ 0.5 %. This makes the imputed data set very impractical for GS simulation studies since the effects of markers with extremely low MAF are difficult to estimate. If markers with a MAF < 1 % are removed from the imputed data set, only 818 (9,154-8,336) supplementary markers can be obtained with BEAGLE 4.0, with respect to the marker data set. The high number of markers with very low MAF in the imputed data set might be a result of the poor degree of genetic relationship between the marker data set and the reference panel. Indeed, only two individuals of the reference panel shared recent common ancestry with the 352 individuals associated with the marker data set [23, 24].

**Fig. 1:**
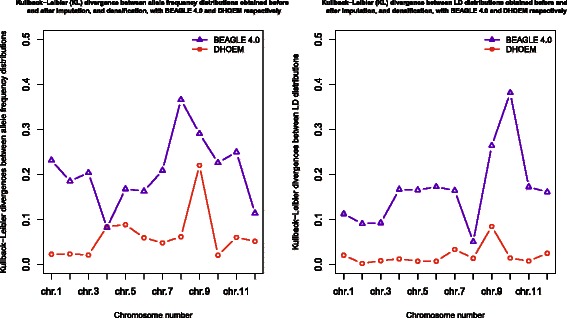
KL divergence between allele frequency distributions and LD distributions obtained before and after imputation and densification, with BEAGLE 4.0 and DHOEM respectively, for chromosomes 1 to 12

The densified marker data set obtained with DHOEM had 16,459 SNP with a MAF ≥ 1 %. DHOEM allows the user to control the MAF limit and this makes the software very practical for simulation studies. The marker data set and the densified data set had 7,879 SNP in common. The Kullback-Leibler (KL) divergence was used to compare the dissimilarity between the distributions obtained from the marker data set and those obtained from the data generated by the two softwares. For each chromosome, Fig. 1 shows the KL divergences between allele frequency distributions and LD distributions, obtained before and after imputation and densification with BEAGLE 4.0 and DHOEM respectively. The KL divergences were calculated using the entropy package [25].

**Fig. 2:**
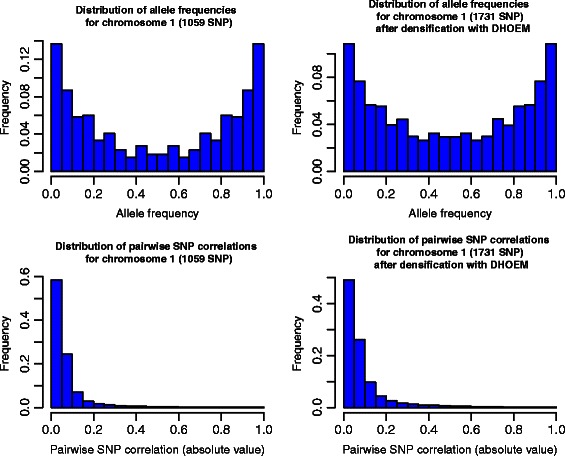
Distributions of allele frequencies and pairwise SNP correlations before and after densification for chromosome 1

**Fig. 3:**
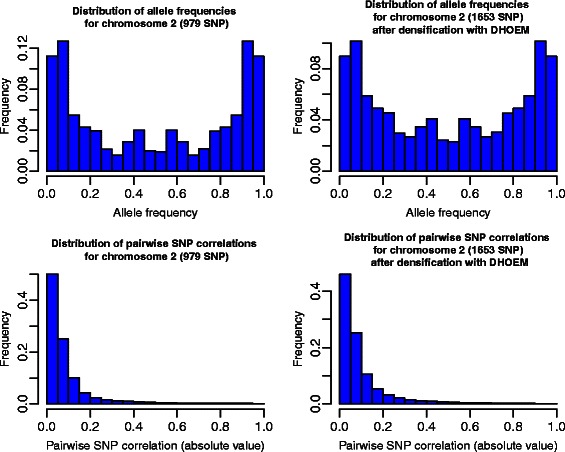
Distributions of allele frequencies and pairwise SNP correlations before and after densification for chromosome 2

In Fig. 1 the KL divergences between the distributions are lower for DHOEM compared to BEAGLE for most chromosomes. This means that the observed distributions obtained from the marker data set are closer to the distributions obtained from the densified data set than the ones obtained from the imputed data set. This is not surprising since there is a close connection between the minimization of KL divergence and maximum likelihood estimation theory. For example, Figs. 2 and 3 illustrate the distributions of allele frequencies and pairewise SNP correlations (i.e. LD), for chromosome 1 and 2, before and after densification of the marker data set with DHOEM. The histograms were drawn using the HistogramTools package [26].

As can be seen in Figs. 2 and 3, there seems to be a good persistence of the initial data structure in the densified marker data. This persistence was evaluated for each chromosome and is discussed in the following subsection on the limits and advantages of DHOEM.

### Limits and advantages of DHOEM

**Fig. 4:**
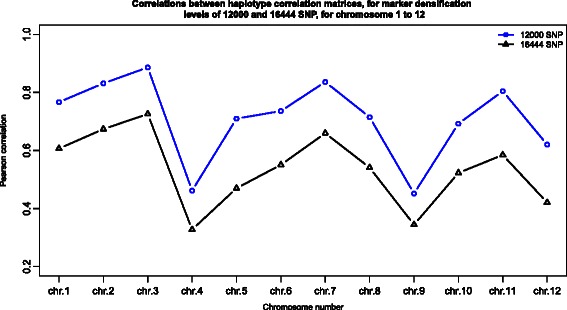
Correlations between haplotype correlation matrices, for chromosome 1 to 12, for marker densification levels of 12000 and 16444 SNP

The persistence of the initial marker data structure in the densified marker data set was evaluated by performing, for each chromosome, a Mantel test of the correlation between the 2*N* by 2*N* haplotype correlation matrices, obtained before and after densification of the marker data set. Persistence was also evaluated for a marker densification of at least 12,000 SNP. For both simulations, the mantel tests were carried out with 10,000 permutations and the p-values obtained for all chromosomes were <10^−16^ which led to the rejection of the null hypothesis of a random correlation. For each chromosome, Fig. 4 shows the correlations between the haplotype correlation matrices at the two marker densification levels.

Figure 4 shows a general decrease in the Pearson correlation for all chromosomes, with an increase in marker density from 12,000 to 16,444 SNP. The average correlations, across all chromosomes, for marker densification of 12,000 and 16,444 SNP are respectively 0.71 and 0.54. This reveals an essential property of DHOEM: too high marker densification using only a small number of available marker data, may ultimately simulate data structures that deviate substantially from what can be observed. For example, the distribution of allele frequencies for each chromosome will approach the theoretical beta distribution, inferred by maximum likelihood, if too high marker densification is applied to a small quantity of marker data.

Clearly, DHOEM is a data dependent procedure that relies on the amount and quality of available data. For instance, the lowest correlations in Fig. 4 were obtained for chromosomes 4 and 9, for which there were a high number of SNP with a low MAF in the initial marker data set. Indeed, 47 % of the total number of markers (i.e. 722 SNP) on chromosome 4, and 57 % of the total number (i.e. 495 SNP) on chromosome 9, had a MAF < 5 %. Hence, for chromosomes 4 and 9, a moderate number of SNP with a moderate MAF were available for the statistical estimation procedures defined in DHOEM. This shows that if a limited amount of data is available, users should proceed with caution when using DHOEM, depending on the objective of their simulation studies.

For example, DHOEM could be useful in pedigree based gene-dropping simulations, where an insufficient amount of marker data prevents building a reliable genomic relationship matrix at the end of each gene-dropping procedure. Indeed, in [27] the additive relationship matrix was built using only pedigree information, as marker data were limited in their gene-dropping simulations. On the other hand, the benefits of using DHOEM might be complicated for QTL mapping simulation studies if not enough marker data are available to represent the real LD structure in a population.

The main advantages of DHOEM emerge in two types of situations; those for which the evolutionary history of a population cannot be ascertained, and those where no representative reference panel is available for the target population under study. For example, the synthetic population described in [23] has a complex evolutionary history that cannot be ascertained, mainly due to long human selection pressure and non-random mating schemes. Hence, in this case it would be tedious, and difficult to use forward-time approaches to simulate data for comparison with DHOEM. Imputation based methods are reliable for increasing marker density if a representative reference panel is available. However, this is not always the case as shown by the imputation results, in our comparison of BEAGLE 4.0 with DHOEM.

## Conclusions

We have presented DHOEM, a simulation tool that exploits real data characteristics to simulate markers that mimic real ones in terms of allele frequencies and LD. DHOEM is a user-friendly tool that allows the user to specify the desired marker density, with a user defined MAF limit, which is produced in a reasonable computation time. Moreover, any method and software, such as those described in [18] for example, can be used to phase unphased genotype data as input for DHOEM as long as DHOEM file formats are respected. However, DHOEM is a data dependent procedure and it may therefore suffer from the amount and quality of available data, and the increase in marker density applied to a marker data set. Depending on the objective of the simulation study, a reasonable tradeoff between the amount of initial data and increase in density applied to the latter should therefore be sought. Nevertheless, by simulating new markers from available real marker data, we believe that DHOEM will help simulation studies in quantitative genetics and breeding, by reflecting results that are to some extent closer to reality than those in simulations that ignore real data characteristics.

## Availability and requirements

**Project name:** DHOEM**Project home page:** http://dhoem.sourceforge.net/ **Operating system(s):** Microsoft Windows**Programming languages:** R**Other requirements:** the software depends on the R packages: MASS, lattice, bisoreg, stats, stats4 **License:** None**Any restrictions to use by non-academics:** None
